# Robotic Surgery in Otolaryngology and Head and Neck Surgery: A Review

**DOI:** 10.1155/2012/286563

**Published:** 2012-04-10

**Authors:** Caio M. Oliveira, Hiep T. Nguyen, Alberto R. Ferraz, Karen Watters, Brian Rosman, Reza Rahbar

**Affiliations:** ^1^Head and Neck Surgery Department, University of Sao Paulo School of Medicine, Sao Paulo, SP CEP 1246903, Brazil; ^2^Department of Urology, Harvard Medical School, Boston, MA 02115, USA; ^3^Department of Otology and Laryngology, Harvard Medical School, Boston, MA 02115, USA; ^4^Department of Otolaryngology and Center for Aerodigestive Disorders, Children's Hospital Boston, MA 02115, USA

## Abstract

Recent advancements in robotics technology have allowed more complex surgical procedures to be performed using minimally invasive approaches. In this article, we reviewed the role of robotic assistance in Otolaryngology and Head and Neck Surgery. We highlight the advantages of robot-assisted surgery and its clinical application in this field.

## 1. Introduction

Recent advances in equipment and surgical techniques have made minimally invasive surgery (MIS) a well-tolerated and efficient technique in several fields of surgery. It has several advantages over standard surgical approaches, including more rapid recovery, lower rate of postoperative infection, decreased pain, better postoperative immune function, and cosmetic results [1–3]. In this way, robotic-assisted surgery (RAS) has gained popularity in several surgical specialties and many institutions are now investing in medical robotic technology for applications in general, urological, cardiac, gynecological, and neurological surgery. This new and exciting technology has been shown to be safe, have better or comparable outcomes, and can be cost effective when compared with conventional surgical approaches [1–3]. This has raised interest in its use in other surgical fields, such as otolaryngology and head and neck surgery.

Head and neck and several airway procedures have been associated with a large amount of surgical dissection with associated large surgical incisions. This can result in major tissue damage, functional impairment, and a decreased quality of life [4]. However, with minimally invasive approaches, the improved video imaging, endoscopic technology, and instrumentation has provided the surgeon with multiple endoscopic access points. While the advance of endoscopic technology has increased surgeon capabilities, the technique still has several challenges associated with it. Examples include: (1) the limited range and degree of motion of instrumentation, (2) operative field limited to “line of sight” (3) lack of three-dimensional imaging of the operative field (4) amplification of physiologic tremors, (5) compromised dexterity and (6) mismatched hand-eye coordination [5, 6]. With these challenges in mind, the development of surgical robotics was rooted in the desire to overcome the limitations of current endoscopic technologies and to expand the benefits of MIS [7].

## 2. The Evolution of the Current Robotic System

 The first robotic surgical system developed was the Puma 560, which was used in 1985 to perform neurosurgical biopsies with increased precision. Since this time, a series of robots have been developed. However, the only FDA approved and actively marketed system (2009, for Transoral Robotic Surgery—TORS [8]) for head and neck surgery is the da Vinci Surgical Robot (Intuitive Surgical Inc., Sunnyvale, CA, USA).

This system has its roots in the National Aeronautics and Space Administration's (NASA) desire to develop a method to provide surgical care to orbiting astronauts via telepresence surgery. [7, 9] Interest in this technology came from both the Stanford Research Institute and the US Army, which saw promise in bringing the technology to the battlefield to provide surgical care to a wounded soldier as soon as possible—even with the surgeon operating remotely. Thereafter, in 1995, the Intuitive Surgical Corporation was set up to produce telerobotic systems for commercial public use, where it was first used in general surgery. Cadiere et al. [10] reported the first two cases of robot-assisted fundoplication in 1999, and Weber et al. [11] reported the first robot-assisted colectomy in 2002. The first robotic surgery performed transorally in the head and neck was carried out in 2005 by MacLeod and Melder [12] whereby a vallecular cyst was excised. In 2006, three patients with tongue base tumors underwent TORS as part of prospective clinical trial by O'Malley Jr. et al. [13].

## 3. The Current Robotic System

**Figure 1:**
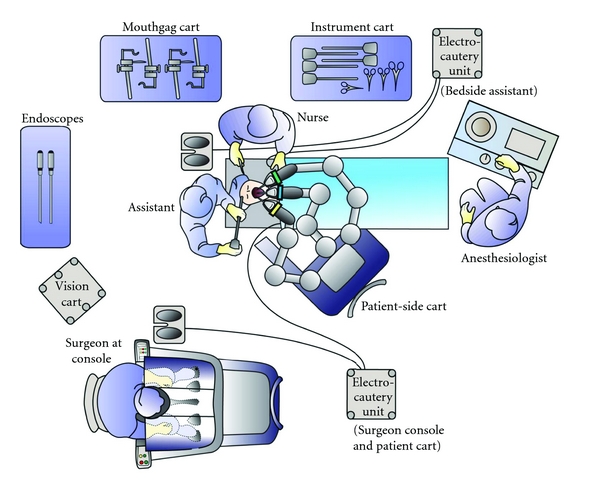
Operation room setup (Courtesy of Intuitive Surgical Inc., 2010).

At its core, the Intuitive Surgical Corporation system is a comprehensive master-slave arrangement, with the surgical robotic cart containing multiple manipulation arms that are operated remotely from a console. The robot contains video-assisted visualization and computer enhancement and is composed of three components: the surgical cart, the vision cart, and the surgeon's console (Figure 1).

The surgical cart (or slave unit) is equipped with four arms; one arm holds a 0° or 30° 12 mm stereoscopic camera (with 2 optical channels, each 5 mm), and the other three arms hold 5 mm (pediatric size) or 8 mm (conventional) EndoWrist instruments (Intuitive Surgical Inc.), that are easily interchangeable by surgical staff according to the surgeon's desire and procedure requirement.

The vision cart is equipped with two light sources, an insufflator, and hardware that generates the three-dimensional image. The cart usually holds another monitor for the assistant surgeon.

The surgeon's console (or master unit) displays two images, one for each eye. This creates a 3-dimensional image that greatly improves depth perception within the surgical field. In addition, the console is the interface for the surgeon to control the instrument, by controlling the hand manipulators. The surgeon's console is equipped with pedals to control the camera and instrument arm clutching (disengagement of the hand controllers from the surgical arms) camera controller, focus adjustment, and electrocautery. There are also surgeon personalization and settings controls.

The EndoWrist instruments are controlled by the surgeon at the master console and provide multiple degrees of freedom, including pitch, yaw, and roll plus two additional degrees of freedom in the wrist and two others for tool actuation—a total of seven degrees of freedom in all. This is in comparison to endoscopic instruments that have just 4 degrees [7]. 

## 4. Advantages of Robot-Assisted Surgery

### 4.1. Enhanced Visualization

The 3-dimensional visualization and tenfold magnification of the operative field enhance the depth of the field and the clarity of the tissue planes during dissection [14]. This can be especially helpful during head and neck surgery and pediatric surgery, because of the small size of the surgical field and the inability to maneuver the instruments and the camera within it. It can also help in distinguishing tissue types in oncological dissection [15].

### 4.2. Elimination of Physiologic Tremors and Scale Motion

The surgical system eliminates the surgeon's tremor through hardware and software filters. In addition, movements can be scaled, whereby large hand movements can be translated into micromovements inside the operative field, allowing the surgeon more precision [1].

### 4.3. Multiarticulated Instruments

EndoWrist instruments have 7 degrees of freedom, which improves dexterity, allowing maneuverability that approaches that of open surgery.

### 4.4. Fatigue Reduction

During the robotic portion of the surgery the surgeon is sitting with his/her forearms resting comfortably on a pad and the head resting against the console, therefore improving ergonomics. This results in reduced body fatigue. With the surgeon sitting at a remote workstation, it eliminates the need to physically twist and turn in awkward positions to move instruments within the operative field while simultaneously visualizing a monitor. In addition, hand muscle fatigue is reduced, which when considered together with improved visualization, makes tasks such as suturing substantially easier. Studies suggest (Berguer and Smith [16]) that robotic surgery is less stressful for the surgeon.

### 4.5. Restore Proper Hand-Eye Coordination

The robotic system eliminates the “fulcrum effect” [17] of endoscopic surgery and makes instrument and camera manipulation more intuitive, emulating another property of open surgery.

### 4.6. Telesurgery

Since the inception of robotic surgery, the wish to overcome geographical constraints and the availability of specialists was an important goal. Marescaux and collaborators [18] described the feasibility and safety of a robot-assisted laparoscopic cholecystectomy at distance using high-speed connection between the surgical unit at Strasbourg, France, and the surgical console in New York. Telesurgery allows for these barriers to be overcome as well as offering new teaching and tutoring possibilities.

### 4.7. Training

The robotic system provides some interesting tools and opportunities for teaching. An experienced surgeon can use another console next to the trainee, which can be activated to command the main arms or auxiliary arms. The Vinci Skills Simulator (Intuitive Surgical Inc.) can be attached to the console, allowing a virtual training environment to be creating while maintaining the same robotic interface [19]. However, there are currently no standardized residency curriculums that formally support the teaching of robotic surgical skills [20].

## 5. Disadvantages of Robot-Assisted Surgery

### 5.1. Absence of Tactile and Haptic Sensation

The surgeon is unable to feel tissue resistance or how tight a knot is being tied. This can lead to ripping of the tissue or the suture. This can be a significant problem early on, although the improvement in visualization is such that the surgeon rapidly learns visual clues to compensate for his lack of feedback. Despite this, RAS still requires careful handling of tissues by the surgeon.

### 5.2. Equipment Size and Weight

Increased physical space requirements in the operating room are needed to accommodate the large and heavy equipment. Additional time and personnel are needed to set it up, along with specialized training for OR staff.

### 5.3. Cost of the Device

Initial installation cost ranges from 1.5 to 2.5 million dollars (US) depending on the model, along with an approximately 100,000 dollars annual maintenance fee and 2000 dollars per instrument (each instrument has a ten use lifespan); the da Vinci robotic system is one of the most expensive operating tools available, making it impractical for many institutions.

### 5.4. New Technology and Unproven Benefit

Stronger studies are needed to assess the real cost-benefit of this technology compared to other techniques.

## 6. Surgical Set-Up

 The description below applies to the TORS procedures, although not all procedures in the head and neck region use this approach. (Other approaches are commented on in each procedure description.)

 Transoral Robotic Surgery (TORS) is defined as surgery performed via the oral cavity that uses a minimum of three robotic arms and allows bimanual manipulation of tissues [21]. It was first developed by Weinstein and O'Malley, who have assessed the feasibility of this technique using the da Vinci Robotic System [13, 22–27]. 

 To minimize obstruction and maximize the communication between the surgeon and his/her assistants in TORS surgery, the surgeon's cart should be located at the end of the operating room, allowing free space to maneuver the surgical cart that is placed on the right side of the patient, opposite to the surgeon. The support staff and instrument carts are located on the side of the patient, opposite the surgeon as well. The anesthesia machine and anesthesiologist are at the patient's foot (Figure 1).

Anesthesia induction is usually done without moving the patient; this technique is described in detail by Chi et al. [28]. According to Chi et al., this method of organization slightly complicates the induction, but vastly simplifies setup for procedure, saving 15–20 minutes per case. Performing the induction across from the anesthesia unit does not require the disconnection/reconnection of IV lines, monitor devices, or the anesthesia circuit, avoiding entanglement with the robotic equipment. Next, with the patient in supine position, the airway is secured via standard endotracheal intubation and the tube is appropriately secured. Safety goggles and a molded dental guard are used to protect the patient.

**Figure 2:**
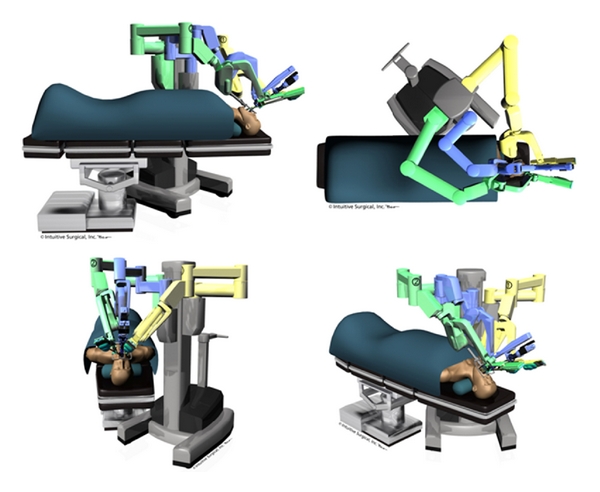
Patient-side cart and robotic arms positioning. (Courtesy of Intuitive Surgical Inc., 2010.)

**Figure 3:**
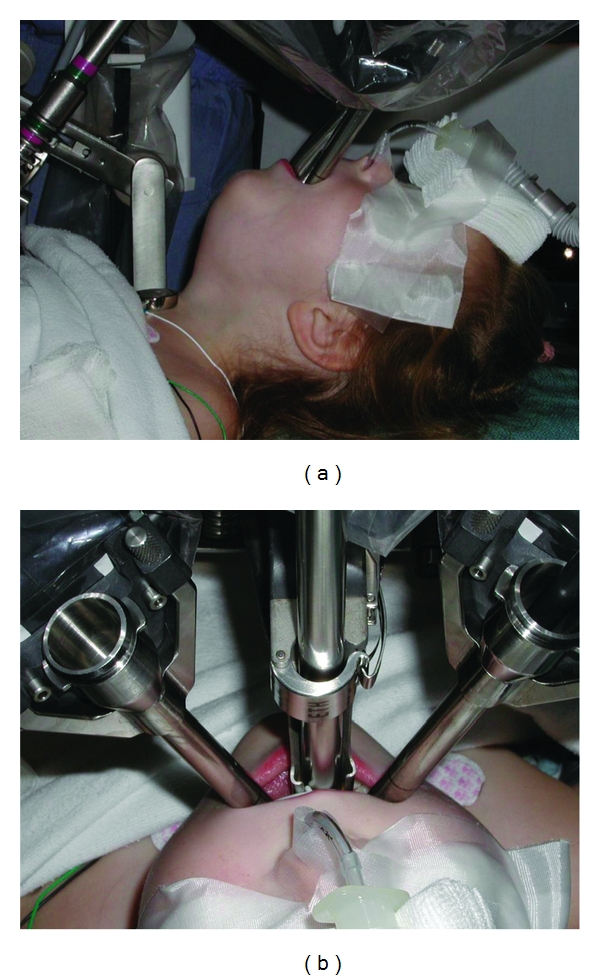
Robotic arms positioning in the oral cavity. Laryngeal reconstruction in pediatric patient. (Courtesy of Dr. Rahbar, 2007.)

 Following induction, the robotic cart is brought in to the right of the patient, and the endoscopy tower and scrub table are brought in to the left. The surgeon then places a retractor in the patient's mouth to gain surgical exposure, and the 3 sterilely draped robotic arms are placed in surgical position (Figures 2 and 3).

## 7. Clinical Applications of Robotic Surgery in the Otolaryngology and Head and Neck Surgery

### 7.1. Head and Neck Oncology (TORS)

O'Malley Jr. et al. initiated the TORS studies in canine and cadaveric models [13, 22–27] and applied the technique to clinical practice. In 2006, three patients underwent robot-assisted transoral tongue base resection in a prospective clinical trial [13]. In this study, the robot enabled the surgeons to easily identify the glossopharyngeal, hypoglossal and lingual nerves, as well as the lingual artery. One T1 and one T2 squamous cell (AJCC cancer staging [29]): two instances of squamous cell carcinoma (one T1 and one T2) were adequately resected with negative margins, good hemostasis, and no postoperative complications.

The different retractor types were assessed first during the cadaveric part of the study, and then at the beginning of each procedure performed in patients. The FK retractor achieved the best (versus Crowe Davis and Dingman retractors) tissue exposure and retraction. The same group published another study in which robot-assisted tonsillectomy was performed on 27 patients with squamous cell carcinoma. 25 of the 27 patients had negative cancer margins and 26 of the 27 patients were able to swallow postoperatively [27].

In 2007, Solares and Strome [30] described transoral carbon dioxide (CO_2_) laser robotic-assisted supraglottic laryngectomy in a 74-year-old woman with a large supraglottic tumor. Postoperatively, the patient was able to swallow by day five. The use of the carbon dioxide laser linked to the surgical robotic system allows more maneuverability of the instrument's tips and improves beyond “sight of beam” limitations. In addition to tumor resection, robotic surgery can be used in the reconstruction of postresection defects. Mukhija et al. reported two cases of robotic-assisted free flap reconstruction in the oral cavity and oropharynx. These studies highlight the improved visualization provided by RAS, avoiding the need to perform a mandibulotomy for access, thereby reducing morbidity and operative time [31]. 

After preliminary studies assessing the feasibility of TORS for oncologic resection, a series of studies were performed to examine the functional outcomes of these procedures [8, 15, 32–39]. Most studies primarily report on oropharyngeal and oral cavity cancer, however, there are also case series on hypopharyngeal and laryngeal malignancy treated with TORS.

Failure due to suboptimal access has been reported. In the study performed by Weinstein et al. [34] in 2010, only 3 of 47 patients were converted to open surgery after attempts failed to reach adequate exposure for resection. Predictors of a difficult access include: retro- and micrognathia and trismus. Other studies demonstrate comparable case exclusion rates, such as Boudreaux et al. [32], who reported 3 of 29, and Iseli et al. [33] found 5 of 54. Moore et al. [8] reported no case exclusion due to unsuitable access. A comprehensive panendoscopy prior to scheduling patients for TORS can identify unsuitable patients and thus reduce surgical risk [40].

Weinstein et al. [34] also report a successful swallowing rate of 97.6% at 12-month followup, while Boudreaux et al. [32] found 79% at last follow up (3 months), and Iseli's study [33] found 83% (12 months of followup). Moore et al. [8] report that all patients returned to normal swallowing (followup time ranged 3 months to 2 years). Predictive factors of poor swallowing following robotic resection included: higher TNM stage, preoperative nasogastric feeding requirement, tumor site (oropharyngeal or laryngeal), and recurrent or second primary tumor resection [33].

Regarding the overall procedure time, we observed a trend to faster procedure times as more cases were being performed. Lawson et al. [15] assessed the robotic learning curve for procedures in the head and neck and found that both set-up and operative times showed a reduction in time as more procedures were performed. For the operative segment, time was reduced from 88 ± 53 to 47 ± 29 minutes. For the overall procedure, time was reduced from 117 ± 64 to 66 ± 33 minutes. However, the time taken for exposure was not reduced with experience.

Another important outcome to consider is recurrence rate after TORS. Although there are no studies assessing recurrence rates at 5 years, preliminary outcomes have been encouraging. In Weinstein's report of advanced oropharyngeal carcinoma, regional control was obtained in 96% and distant control in 91% of cases at 18 months follow up [34]. Accordingly with Machtay et al. [41], local control was always achieved if negative oncological margins were obtained. The robot can thus provide an excellent approach to cancer, improving the ability to interpret the adequacy of the resection margins—an important factor in determining whether adjuvant therapy is indicated [42]. Further studies are needed to assess the short- and long-term outcomes of TORS when compared to other more established techniques Table 1.

### 7.2. Benign Head-Neck (TORS)

The first published clinical application of TORS, performed by MacLeod and Melder, was marsupialization of a vallecular cyst [12]. Vicini et al. assessed the effectiveness of robot-assisted surgery in Obstructive Sleep Apnea-Hypopnea Syndrome (OSAHS) [44, 45]. In these studies, 20 patients underwent a tongue base resection, with some patients also having a supraglottoplasty and uvulopalatoplasty performed. Overall patient satisfaction, assessed by a Visual Analogue Scale (VAS, 0 to 100%) was 94%. A reduction in the Epworth score (mean ESS improvement was 5.9 + 4.4 SD) and Apnoea-Hypopnoea Index was seen (mean AHI improvement was 24.6 + 22.2 SD). All patients were decannulated between day 4 and 13 after surgery and regained a satisfactory ability to swallow within 2 weeks. No operative or postoperative complications (10 months of followup) were seen. This study showed the feasibility and safety of robotic tongue base resection techniques. 

Another cadaveric study conducted at the University of Pennsylvania in 2010 by Lee et al. showed the feasibility of transoral approach for decompression of the craniocervical junction, demonstrating the possible use of the robot in the future for conditions such as compression for basilar invagination, congenital skull base malformations, extradural lesions, and skull-base tumors [46].

### 7.3. Robot-Assisted Thyroidectomy

 The transaxillary robotic technique was first described in 2005 by Lobe et al. [47], where a hemithyroidectomy was successfully performed in a pediatric patient. In 2008, the same group reported a bilateral axillary approach for total thyroidectomy in two pediatric patients [48].

 In adults, the largest experience in robot-assisted thyroidectomy by Kang et al. who developed the gasless transaxillary technique [49, 50] and reported a series of 338 patients. In 2009, a case control study of 41 robotic cases and 43 conventional thyroid surgery patients was reported [51, 52]. Unlike the transoral technique described previously, this procedure dissects a tunnel on the anterior surface of the pectoralis major muscle and clavicle by electrocautery under direct vision, before the robotic portion of the surgery. With the patient-placed supine under general anesthesia, the neck is slightly extended, and the ipsilateral arm is abducted at the shoulder to minimize the distance between the axilla and neck. A second incision is made on the medial side of the anterior chest wall to insert the 4th robot arm that will be used for thyroid retraction, and it is connected to a continuous suction system. The dissection is approached through the avascular space of the sternocleidomastoid muscle branches and beneath the strap muscle until the contralateral lobe of the thyroid is exposed. Next, the operation proceeds in the same manner as a conventional open thyroidectomy. Two 8 mm instruments are introduced through the breast incision, and the 3rd arm carries the 12 mm endoscope [51]. Robotic thyroidectomy using a transaxillary approach leaves a scar in the axilla that is covered by the patient's arm. This is important when we consider that thyroid disease is more common in women, and the incidence is increasing in young women, raising concerns about cosmetic results [53]. 

 Robotic-assisted thyroidectomy has been associated with a lower degree of postoperative discomfort, a higher degree of patient cosmetic satisfaction, and subjective improvements in swallowing discomfort, when compared to the conventional surgery [51–53]. A few cases of recurrent laryngeal nerve injury have been reported. In 2011, Lee et al. published a multicenter retrospective study of 1,043 cases of low-risk differentiated thyroid carcinoma and compared the results of robotic-assisted thyroidectomy to laparoscopic and open thyroidectomy surgical series. This study supports the statement that robotic use is safe, feasible, and provides the similar outcomes to other techniques, while also overcoming their limitations [54]. In addition, it seems that the indication for robotic thyroidectomy can be expanded to include advanced thyroid cancer, because lymph node resection can be performed with great dexterity, removing a similar number of lymph nodes as in open surgery. Other groups have reported slight modifications to this technique. Tae et al. [55] inserted the 4th arm trocar through an ipsilateral periareolar nipple incision, while Lee et al. [56] used a bilateral transaxillary approach with CO_2_ insufflation. In any case, these techniques were shown to be feasible and have comparable results to open surgery, although CO_2_ insufflation has been associated with increased probability of pneumomediastinum and air embolism [57] Table 2. 

### 7.4. Robot-Assisted Parathyroidectomy

Technically similar to the surgery performed for thyroidectomy, robot-assisted parathyroidectomy was described in 2004 by Bodner et al. [59–62]. This technique involves a 5-to-6 cm vertical skin incision in the axilla with a subcutaneous skin flap created from the axilla to the anterior neck area over the pectoralis major muscle and clavicle under direct vision. An external retractor attached to a lifting device maintains the working space. A second 0.8 cm skin incision is made on the anterior chest. With these 2 incisions, 4 robotic arms can be inserted—3 in the axilla and 1 in the anterior chest wall. 

Following this study, other publications detailed further robot-assisted parathyroidectomy [63–69]. The most recent and largest study Tolley et al. included 11 patients with hyperparathyroidism [70]. This study showed that the robot-assisted surgery allowed adequate visualization of important anantomicanatomic structures in this region, good resection, and a hospital length of stay comparable to nonrobotic minimally invasive surgeries [71–77]. Only one case needed to be converted to open surgery due to the patient's large body habitus—a factor shown to be a predictor of longer operative times [70]. Validated questionnaires regarding quality of life and cosmetic appearance showed good subjective results for this new approach. 

### 7.5. Skull Base Surgery

 The fundamental studies that established the technical feasibility of TORS to gain access to many regions, such as the oral cavity, oropharynx, hypopharynx, and larynx, raised the question about whether the robot can reach more difficult places. TORS used in skull base surgery was initially assessed by O'Malley Jr. and Weinstein [78], using animal and cadaver models. They also reported the first human case—a patient that underwent resection of parapharyngeal cystic neoplasm extending into the infratemporal fossa. Overall there were no adverse surgical events. Concern regarding identification of important structures, such as the carotid artery, jugular vein and cranial nerves was raised, and was solved by appropriate demonstration of surgical technique and hemostasis. 

In 2010, another study performed by O'Malley Jr. and Weinstein assessed the outcomes of 10 patients undergoing parapharyngeal space resection using the TORS approach. The surgery was performed in 9 of the 10 patients, with acceptable operative time and blood loss, and no significant complications such as hemorrhage, infection, trismus or tumor spillage. One patient was converted to an open transcervical approach due to difficulties found during resection and to avoid the risk of tumor spillage. In 7 patients that had resection of a parapharyngeal space pleomorphic adenoma, local control was obtained in all 7 patients, although tumor spillage was reported in one patient. The TORS approach was found to offer reduced complication rates when compared to the transcervical approach [79, 80]. 

Another approach to the infratemporal fossa was developed by McCool et al. [81], in which 6 complete and 2 partial resections were performed using a suprahyoid port, while the other arms were placed transorally. In another report, Hanna et al. [82] obtained excellent access to the anterior and central skull base in cadavers, including the cribriform plate, fovea ethmoidalis, medial orbits, planum sphenoidale, nasopharynx, pterygopalatine fossa, and clivus. In addition, sella turcica and suprasellar and parasellar access was achieved using the robotic arms. However, there is a continuing need for further development of appropriate instruments, in terms of size, flexibility, and function.

### 7.6. Pediatric Surgery

Although there are studies of robotic surgery thyroidectomy in children [47, 48], which we have discussed previously, studies of robotic surgery in the pediatric population are sparse. To date, the only pediatric case series is that described by Rahbar et al. [83] in 2007 at Children's Hospital Boston. In this study, 4 pediatric cadaver larynxes were used to assess precision and tissue handling using a robotic-system. 5 living patients were enrolled to undergo a laryngeal cleft repair. Equipment size was the main limiting factor for these procedures, resulting in limited transoral access in 3 of 5 the patients. The other 2 patients, who had type 1 and type 2 laryngeal clefts, had successful surgical repairs using the robotic system.

## 8. Conclusion

 The trend towards the use of minimally invasive surgery has had an impact on the way new technology is thought of, developed, and incorporated into clinical practice. Robotic surgery is continuing to advance, and is overcoming its limitations. It is improving the outcomes, such as reducing hospital stays and infection rates, and allowing for better cosmetic results. However, surgical robots were developed to perform procedures in spacious cavities, such as the abdomen, and thus, the instruments are over sized to perform many of the otolaryngology and head and neck procedures. The da Vinci robot system is starting to be adopted to carry out a number of otolaryngology procedures, and it has done so with excellent results so far. 

Other limitations of robotic surgery are like the large size of the robotic system, which necessitates additional manpower to set it up and creates new challenges for the anesthesia team and surgical assistants. Unfortunately, the high cost of the robotic equipment forbids its routine presence and use in most operating rooms across the globe. This calls for the development of smaller, less expensive and easy to operate robotic platforms, which are portable and flexible to use, as well as specific instruments for tasks in head and neck surgery. 

 Besides the evidence of robotic feasibility and safety in head and neck surgery, postoperative outcomes regarding airway management and oropharyngeal function are comparable or better to traditional surgical approaches. Although we did not explore the details concerning oncologic results, robot-assisted surgery showed a trend towards favorable cure and recurrence rates. This can be attributed to its capability to resect tumour en-bloc—a feature that is provided by the increased dexterity and 3D visualization of the robotic system. We believe that future studies comparing robotic techniques to Transoral Laser Microsurgery (TLM), open surgery and chemoradiotherapy are required to support these assertions. Reported studies are supportive of the feasibility and safety of robotic surgery in head and neck procedures and encourage its continuing use and exploration. 

## Figures and Tables

**Table 1:** Major clinical series in oncologic TORS.

Authors	Subjects (no.)	Tumor site/sites approached (no. of patients)	Exclusions due unsuitable access	Blood loss (mL)	Mean surgical time (min)	Average hospital stay (days)	Followup (F/U) time (months)	Ability to swallow	Mean decann-ulation (days)
Boudreaux et al. [32]	29	Oral cavity = 2 oropharynx = 19 hypopharynx = 7 larynx = 1	3	2–150 (mean 51)	99	1–13 (mean 2.9)	3	79% at last F/U	NR
Moore et al. [8]	45	Base of tongue = 26 tonsillar fossa = 19	0	NR	71	1–10 (mean 3.8)	3–24	100% at last F/U	14
Iseli et al. [33]	54	Oral cavity = 6 oropharynx = 33 larynx = 12 hypopharynx = 3	5	NR	NR	1–7	2–24	83% at mean 12 months F/U	5 days
Weinstein et al. [34]	47	Base of tongue = 23 tonsil = 23 soft palate = 1	3	220 (mean)	NR	NR	26 (mean)	97.6% at last F/U	8 days
White et al. [43]	89	Oral cavity = 2 oropharynx = 77 Larynx = 10	0	NR	NR	NR	26 (mean)	100% at last F/U	NR

NR, not reported.

**Table 2:** Major clinical series in robot-assisted thyroidectomy.

Authors	Subjects (no.)	Thyroidectomy type	Technique and approach	Operation time (min)	Tumor average size (cm)	Average hospital stay (days)	Complications (%)
Lee et al. [56]	15	Total = 14 Partial = 1	Bilateral axillo-breast with C02 insufflation	218	<1	3.5	NR
Kang et al. [51]	338	Total = 104 Partial = 234	Gasless transaxillary	144	0.8	3.3	Transient Hypocalcaemia = 41.3% transient RLN paresis = 3.8% Seroma formation = 1.7% permanent RLN injury = 0.8% hematoma formation = 0.6% horner's syndrome = 0.2% Transient brachial plexus neuropraxia = 0.2%
Tae et al. [55]	41	Total = 10 Partial = 31	Gasless axillo-breast and axillary	179	1.63	6.4	Transient hypocalcaemia = 20% seroma formation = 4.9% transient RLN paresis = 2.4%
Lee et al. [54]	1043	Total = 366 Partial = 677	Gasless transaxillary	132	0.8	2.9	Transient hypocalcaemia = 18.4% transient RLN paresis = 4.3% seroma formation = 2% chyle leakage = 1.2% permanent RLN injury = 0.5% hematoma formation = 0.5% tracheal injury = 0.3% transient brachial plexus neuropraxia = 0.3% horner's syndrome = 0.1%
Lee et al. [58]	580	Total = 135 Partial = 445	Gasless transaxillary	126 (partial) 151 (total)	0.58	3.3	Transient hypocalcaemia = 37.8% transient hoarseness = 3.3% permanent RLN injury = 0.7% seroma formation = 2.9% hematoma formation = 0.5% tracheal injury = 0.3% traction injury of ipsilateral arm = 0.2%

RLN, Recurrent Laryngeal Nerve; NR, not reported.
